# A modified stent-free Chen's U-suture technique in pediatric pancreaticojejunostomy: safety and efficacy

**DOI:** 10.3389/fped.2026.1890358

**Published:** 2026-07-21

**Authors:** Houfang Kuang, Jing Zhang, Zhenchuang Zhu, Nannan Zheng, Xufei Duan, Xuan Sun, Xueqiang Yan

**Affiliations:** 1 Department of Hepatobiliary and Oncology Surgery, Wuhan Children’s Hospital (Wuhan Maternal and Child Healthcare Hospital), Tongji Medical College, Huazhong University of Science & Technology, Wuhan, China; 2 Department of Radiology, Wuhan Children’s Hospital (Wuhan Maternal and Child Healthcare Hospital), Tongji Medical College, Huazhong University of Science & Technology, Wuhan, China; 3 Emergency and Critical Care Medical Center, Wuhan Children’s Hospital (Wuhan Maternal and Child Healthcare Hospital), Tongji Medical College, Huazhong University of Science and Technology, Wuhan, China

**Keywords:** children, modified stent-free Chen's U-suture technique, pancreatic fistula, pancreaticojejunostomy, pediatric pancreatic surgery, soft pancreas/small pancreatic duct

## Abstract

**Background:**

Pediatric pancreatic surgery is challenging due to soft pancreatic texture and small main pancreatic duct (MPD) diameter, both risk factors for postoperative pancreatic fistula (POPF). The modified stent-free Chen's U-suture technique, a continuous transpancreatic U-shaped invagination pancreaticojejunostomy without stenting, has shown low clinically relevant POPF (CR-POPF) rates in adults. This study evaluates its safety and feasibility in children.

**Methods:**

From July 2018 to November 2025, five children (2 males, 3 females; aged 4.3–10.1 years) underwent pancreatic resection with the modified stent-free Chen's U-suture technique (no MPD stent placement). Diagnoses included pancreatic head solid pseudopapillary neoplasm (SPN, *n* = 2, one with jaundice), body SPN (*n* = 1), grade III trauma (*n* = 1), and complex iatrogenic injury (*n* = 1). The primary endpoint was POPF rate (ISGPS 2016).

**Results:**

All procedures were successful. Median operative time was 5 h (range 3–8); mean pancreaticojejunostomy time 17.8 min (15–21); median blood loss 50 mL (20–100). Two patients had transient mild hyperamylasemia (< 2×upper limit of normal) with imaging showing localized perianastomotic edema/fluid collection without diffuse parenchymal edema or necrosis, managed conservatively. One patient met the criteria for a biochemical leak; no CR-POPF (0%, 95% CI: 0.0%–52.2%) (grade B/C) occurred. No infection, hemorrhage, reoperation, or readmission occurred. Drains were removed on days 5–14 (median, 7 days). At median 25-month follow-up (range 4–92), one child required pancreatic enzyme replacement for fat malabsorption 4 years post-surgery; no new diabetes or other malabsorption occurred. One patient had asymptomatic mild distal MPD dilatation (2.9 mm) at 25 months; another had normal MPD shape (2.3 mm) at 4 months.

**Conclusion:**

The modified stent-free Chen's U-suture technique demonstrates promising feasiblility and safety for pediatric pancreatic head, neck, or body tumor resection, grade III trauma, and complex iatrogenic injuries, achieving a 0% CR-POPF rate in this small cohort (95% CI: 0.0%–52.2%) without the need for internal stenting. Transient mild hyperamylasemia with localized perianastomotic inflammatory changes is uncommon and manageable. Long-term pancreatic function is generally preserved, though asymptomatic MPD dilatation may occur.

## Introduction

Pancreatic surgery, particularly the resection of tumors in the pancreatic head and neck, surgical treatment of severe pancreatic trauma, or management of severe pancreatic injury, presents a significant challenge in pediatric surgery. The key to surgical success lies not only in the complete resection of the lesion but also in safe and reliable gastrointestinal reconstruction. Among these factors, the quality of pancreatico-intestinal anastomosis is the core determinant of serious complications such as postoperative pancreatic fistula (POPF) (1, 2). Pancreatic fistula can lead to secondary abdominal infections and gastrointestinal bleeding, and is one of the major risk factors for perioperative mortality following pancreatectomy (3).

Compared with adults, the pancreas of children has unique anatomical and physiological characteristics: the main pancreatic duct is slender (often < 2 mm), the pancreatic parenchyma is soft and fragile, and the exocrine function is vigorous. This makes the traditional pancreatic duct-mucosa anastomosis extremely difficult to perform in children, significantly increasing the risk of anastomotic failure and pancreatic fistula (4–6). Furthermore, the 2025 International Association of Pancreatology (IAP) guidelines emphasize that although the diagnostic criteria for acute pancreatitis in children are the same as in adults (i.e., at least two of: characteristic abdominal pain, serum amylase/lipase > 3× upper limit of normal, and imaging findings), their accuracy may be lower due to atypical presentations such as a lower proportion of abdominal pain, and there is a lack of validated definitions for postoperative acute pancreatitis specifically in the pediatric population (7). Therefore, seeking a pancreatic-intestinal anastomosis method suitable for the anatomical and physiological characteristics of children is of great significance for reducing postoperative complications and improving prognosis.

The Chen's U-suture technique (Chen's pancreaticojejunostomy), an invagination pancreaticojejunostomy (PJ) pioneered by Professor Chen Xiaoping's team in 1995, with its core technology using a single needle to pass through the muscle layers of the pancreatic stump and the jejunum to achieve a full circumferential anastomosis. Officially published in 2007, the latest results in 2014 showed an low pancreatic fistula rate of 4.2% (1, 8, 9). This surgical method has been proven in adult pancreatic surgery to significantly reduce the incidence of clinically relevant pancreatic fistula, especially in patients with soft pancreas combined with small pancreatic ducts (diameter ≤ 3 mm), demonstrating good safety and reliability (10–12). However, the clinical effectiveness of this technique in children, especially in cases of pancreatic head and neck tumors, grade III pancreatic trauma, and complex iatrogenic injuries, remains unclear. Moreover, there is no unified consensus internationally and domestically regarding whether pancreatic duct stents should be routinely placed during pediatric pancreatic-intestinal anastomosis: Although stents theoretically can play a role in supporting drainage and reducing pancreatic duct pressure, they also have potential risks such as stent blockage, displacement, injury to the pancreatic duct mucosa, and induction of chronic inflammation (11, 13).

This study retrospectively analyzed the clinical data of 5 children who underwent pancreatic resection combined with modified stent-free Chen's U-suture technique (no MPD stent placed due to the small duct diameter). Our aim was to evaluate the short-term safety and efficacy, as well as the long-term outcomes, of this anastomosis technique in children with specific pancreatic diseases, focusing on the feasibility of performing the procedure without an internal stent and its impact on the occurrence of postoperative pancreatitis and long-term MPD morphology. This study provides a reference for the clinical application of this technique in the pediatric population.

## Patients and methods

### Patients

Clinical data of five children who underwent pancreatic resection combined with modified stent-free Chen's U-suture technique for pancreatic diseases from July 2018 to November 2025 in our hospital were collected. Inclusion criteria: (1) Age ≤ 14 years; (2) Received pancreatic resection due to pancreatic head, neck, and body tumors, pancreatic grade III trauma, or iatrogenic injury; (3) Digestive tract reconstruction with modified stent-free Chen's U-suture technique, and no intraoperative placement of a pancreatic duct stent was left during the operation. Exclusion criteria: Patients with incomplete clinical data or those lost to follow-up.

**Figure 1:**
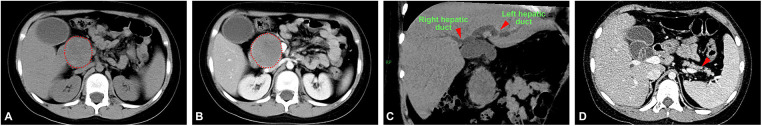
(Case 1): Non-contrast CT shows a round soft-tissue density mass in the pancreatic head (**a**, red circle), which demonstrates moderate enhancement on contrast-enhanced CT (**b**, red circle), accompanied by dilatation of intrahepatic and extrahepatic bile ducts (**c**, red circle at the porta hepatis and red arrowhead at the left and right hepatic ducts), and atrophy of the pancreatic body and tail (**d**, red arrowhead).

**Figure 2:**
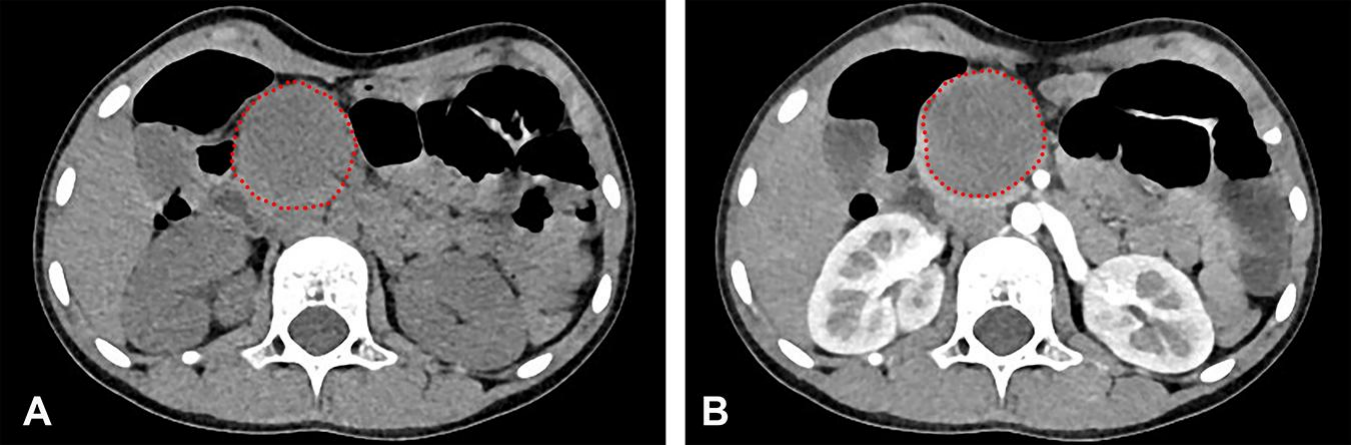
(Case 2): Non-contrast CT reveals a cystic hypodense lesion in the pancreatic head (**a**, red circle); on contrast-enhanced CT, it shows no enhancement with well-defined margins, and the surrounding pancreatic parenchyma exhibits a “ball-holding” sign of envelopment (**b**, red circle). Copyright by Chen Y, et al. [21].

**Figure 3:**
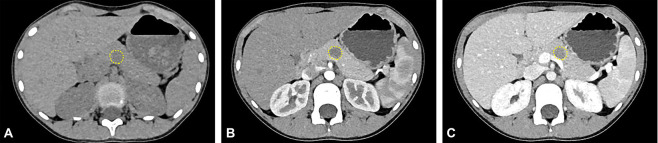
(Case 3): Non-contrast CT demonstrates a slightly hypodense lesion at the junction of the pancreatic body and neck (**a**, yellow circle), which shows progressive enhancement during the arterial phase **(b)** and venous phase **(c)** on contrast-enhanced CT (yellow circle).

**Figure 4:**
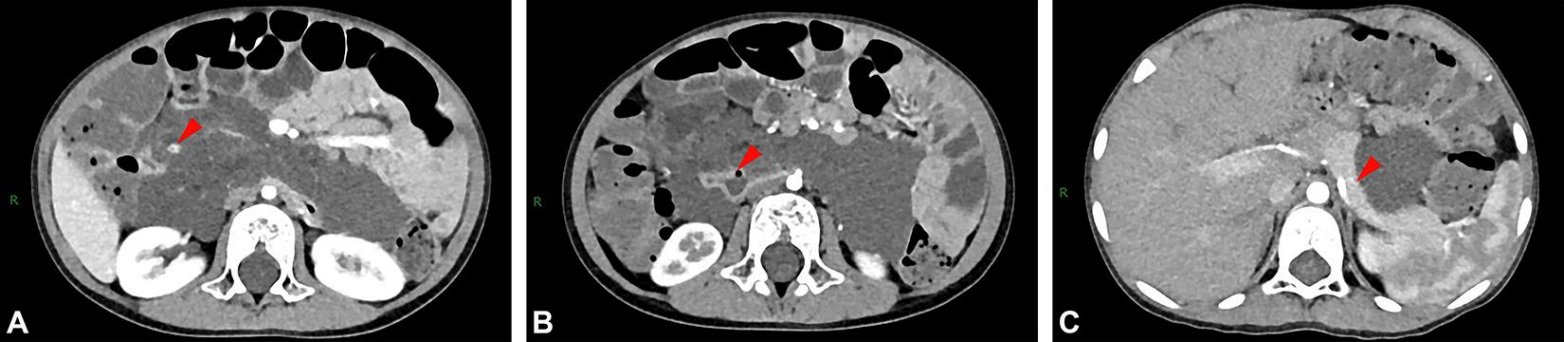
(Case 4): CT reveals a cystic lesion in the right mid-upper abdomen with heterogeneous density, containing multiple linear septations and calcifications (**a**, red arrowhead); the lesion envelops the duodenum (**b**, red arrowhead) and extends anteriorly to the front of the pancreatic body and tail (**c**, red arrowhead), suggestive of a lymphangioma.

**Figure 5:**
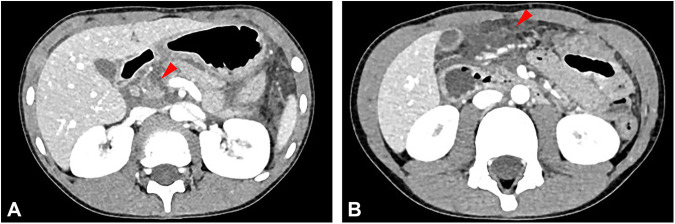
(Case 5): CT shows discontinuity of the pancreatic neck (**a**, red arrowhead), heterogeneous parenchymal density, blurred peripancreatic fat planes, and associated peripancreatic fluid collection (**b**, red arrowhead).

A total of 5 children were included in this group (2 boys and 3 girls), aged from 4 years and 4 months to 10 years and 1 month. Patient demographic and clinical characteristics as shown in Table 1. The cases included: one pancreatic head solid pseudopapillary tumor (SPN) with obstructive jaundice (Case 1/Figures 1A–D); one SPN in the pancreatic head (Case 2/Figures 2A,B); one SPN in the pancreatic body (Case 3/Figures 3A–C); one complex iatrogenic injury (biliary fistula and pancreatic leakage following surgery for mesenteric lymphangioma) (Case 4/Figures 4A–C); and one grade III pancreatic trauma (complete transection of the pancreatic neck) (Case 5/Figures 5A,B). All the children underwent preoperative abdominal contrast-enhanced CT assessment, and the specific imaging features are shown in the following figure.

**Table 1:** Preoperative general information of 5 pediatric cases.

Case	Sex	Age	Symptoms	Physical Findings			Preoperative CT Imaging Findings				
				Jaundice	Tenderness	Peritonitis	Location	Size (mm)	Characteristics of the lesion	Other findings	Pancreatic Duct Condition
1	Male	10 years and 1 month	Generalized pruritus with jaundice	+	+	-	Pancreatic head	54 × 49 × 56	Solid	Dilatation of the common bile duct (8 mm) and the left and right hepatic ducts	Pancreatic tail agenesis
2	Female	6 years and 1 month	Abdominal pain with vomiting	-	-	-	Pancreatic head	44 × 40 × 36	Cystic-solid	-	< 2 mm
3	Female	8 years and 9 months	Abdominal pain with fever	-	-	-	Pancreatic body	12 × 12 × 13	Solid	-	< 2 mm
4^a^	Female	4 years and 4 months	Abdominal mass detected	+	+	+	Pancreatic head	129 × 62 × 113	Cystic	The mass encases part of the intestine, extending partially to the anterior aspect of the pancreatic body and tail	Not visualized
5	Male	7 years and 11 months	Abdominal pain, nausea, and vomiting for one day after blunt trauma to the right upper abdomen	-	+	+	Pancreatic neck	-	Continuous interruption of the pancreatic neck	Peripancreatic, lesser omental sac, peritoneal, and perienteric fluid accumulation	< 2 mm

+, positive finding (e.g., symptom or sign present); -, negative finding (e.g., symptom or sign absent).

a Three days postoperatively, this patient exhibited a large amount of bile draining from the abdominal drainage tube, and a second surgery was performed for suspected bile leakage.

No patients were excluded due to incomplete data or loss to follow-up.

### Surgical technique

**Figure 6:**
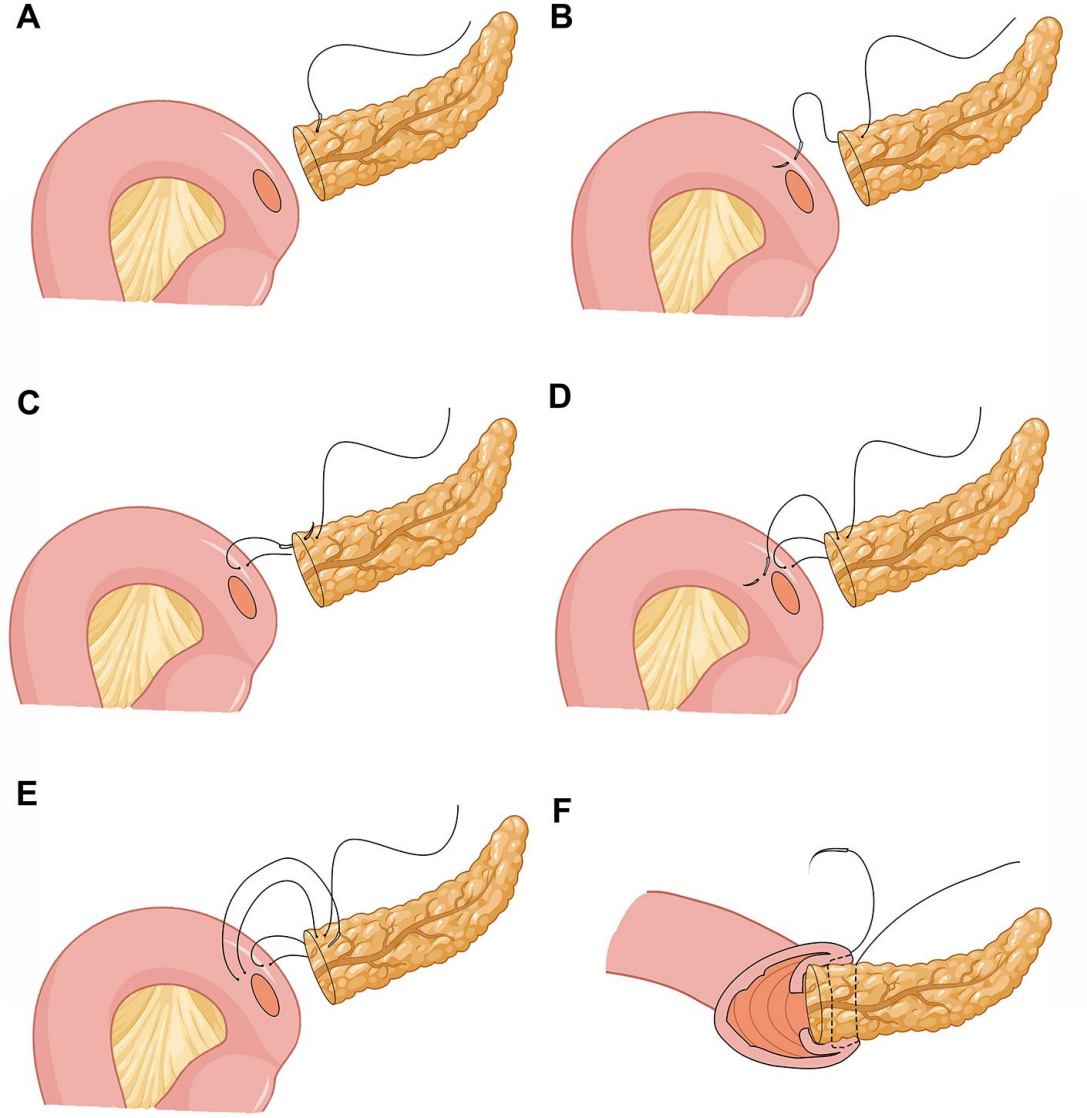
Schematic illustration of the modified stent-free chen's U-suture technique in pediatric pancreaticojejunostomy. **(A)** Step 1: The needle is inserted through the anterior wall and exits through the posterior wall of the pancreas, 10 mm from the pancreatic stump. **(B)** Step 2: The needle and suture are turned toward the jejunum, and the posterior seromuscular layer (5–10 mm from the incision edge) is sutured at the upper edge of the jejunal opening. **(C)** Step 3: Returning to the pancreas, the needle is passed through the posterior wall and exits through the anterior wall. **(D)** Step 4: The anterior seromuscular layer of the jejunum is sutured symmetrically to the needle entry point on the posterior wall. **(E)** Continuous U-sutures are placed sequentially, gradually invaginating the pancreatic stump into the jejunal lumen. **(F)** After tightening the sutures, the intestinal wall naturally encapsulates the pancreatic stump.

All surgeries were performed by the same group of highly experienced pediatric surgeons. The extent of resection was determined based on the nature and location of the lesion:

1. **Tumor resection:** Case 1 underwent radical pancreaticoduodenectomy, Case 2 underwent duodenal-preserving partial pancreatic head resection, and Case 3 underwent robot-assisted laparoscopic central pancreatectomy.
2. **Management of the remnant:** In the patient who underwent central pancreatectomy (Case 3) and the trauma patient (Case 5), the distal pancreatic remnant required pancreaticojejunostomy, while the proximal pancreatic head remnant was suture-closed. In pancreaticoduodenectomy cases (Case 1, 2, 4), no residual pancreatic parenchyma remained on the duodenal side.
3. **Management of iatrogenic injuries and trauma:** Case 4 underwent pylorus-preserving pancreaticoduodenectomy, and Case 5 underwent debridement plus Roux-en-Y distal pancreaticojejunostomy.
4. **Anastomosis techniques** (modified stent-free Chen's U-suture pancreaticojejunostomy):
  (1) **Pancreatic stump preparation:** The stump was dissected for approximately 0.5 cm. No pancreatic duct stent was placed.
  (2) **Jejunal preparation:** A small incision of about 5 mm (not exceeding 10 mm) was made on the antimesenteric side of the jejunum, using the elasticity of the intestinal wall to accommodate the pancreatic stump.
  (3) **U-suture (core step):** Use 3–0 or 4–0 polypropylene non-absorbable monofilament sutures (e.g., Prolene) to minimize tissue reactivity and preserve tensile strength throughout the critical healing period. Begin at the superior or inferior margin of the pancreas and form a complete “U” shape via the following four steps (Figures 6A–D):
    ° **Step 1:** 10 mm from the stump, the needle was inserted through the anterior wall and exited through the posterior wall of the pancreas.
    ° **Step 2:** The needle and suture were rotated to the jejunum, and the posterior seromuscular layer (5–10 mm from the incision edge) was sutured at the upper edge of the jejunal incision.
    ° **Step 3:** Returning to the pancreas, the needle was inserted through the posterior wall and exited through the anterior wall.
    ° **Step 4:** Finally, the anterior seromuscular layer of the jejunum was sutured symmetrically to the needle entry point on the posterior wall.
  (4) **Continuous suture and invagination** (Figures 6E,F)**:** The above steps were repeated to form a row of continuous “U” sutures, gradually pulling the pancreatic stump into the jejunal lumen. After tightening the sutures, the intestinal wall naturally encapsulated the pancreatic stump.

In this case series, pancreatic duct stents were not routinely placed because the main pancreatic duct could not be clearly identified. All children received octreotide routinely for 5 days postoperatively; if pancreatic leakage occurred, the medication duration was extended.

### Data collection

1. **Perioperative indicators:** operative time, pancreaticojejunostomy time, intraoperative blood loss, and postoperative complications (pancreatic fistula, hemorrhage, infection, etc.). The diagnosis of pancreatic fistula follows the 2016 International Study Group on Pancreatic Surgery (ISGPS) criteria: amylase concentration in drainage fluid of any volume on or after postoperative day 3 exceeding three times the upper limit of normal serum amylase, classified by clinical symptoms as biochemical leak, Grade B pancreatic fistula, or Grade C pancreatic fistula. With 0 CR-POPF events in 5 patients, the exact 95% confidence interval for the CR-POPF rate is 0.0%–52.2% (Clopper-Pearson method).
2. **Laboratory indicators:** dynamic changes in complete blood count, CRP, and serum amylase during hospitalization; amylase concentration in drainage fluid of any volume on or after postoperative day 3. Postoperative pancreatitis was defined in accordance with the 2025 IAP guidelines for acute pancreatitis in children, which require at least two of the following: characteristic abdominal pain, serum amylase/lipase > 3× upper limit of normal (ULN), and imaging findings of acute pancreatitis (e.g., focal or diffuse pancreatic parenchymal edema and/or peripancreatic stranding without necrosis) (7). Given the absence of a validated pediatric definition specifically for post-pancreatectomy acute pancreatitis, we applied the IAP diagnostic framework. In this study, transient hyperamylasemia (< 2 × ULN) in the absence of abdominal pain or diffuse parenchymal changes on imaging did not meet the IAP criteria for acute pancreatitis; such events were recorded as self-limited biochemical phenomena rather than clinical postoperative pancreatitis.
3. Time to initiation of oral intake and time to drain removal.
4. Pancreatic imaging examinations.
5. **Long-term follow-up:** via outpatient visits and telephone follow-up, recording children's growth and development, presence of symptoms such as abdominal pain, diarrhea, steatorrhea, polydipsia, and polyuria; measuring fasting blood glucose and glycated hemoglobin (HbA1c); evaluating pancreatic morphology, pancreatic duct diameter, and tumor recurrence using abdominal ultrasound, CT, or MRCP. The last follow-up for this cohort was in February 2026. The median follow-up duration was 25 months (range: 4–92 months). All patients completed full follow-up.

## Results

**Figure 7:**
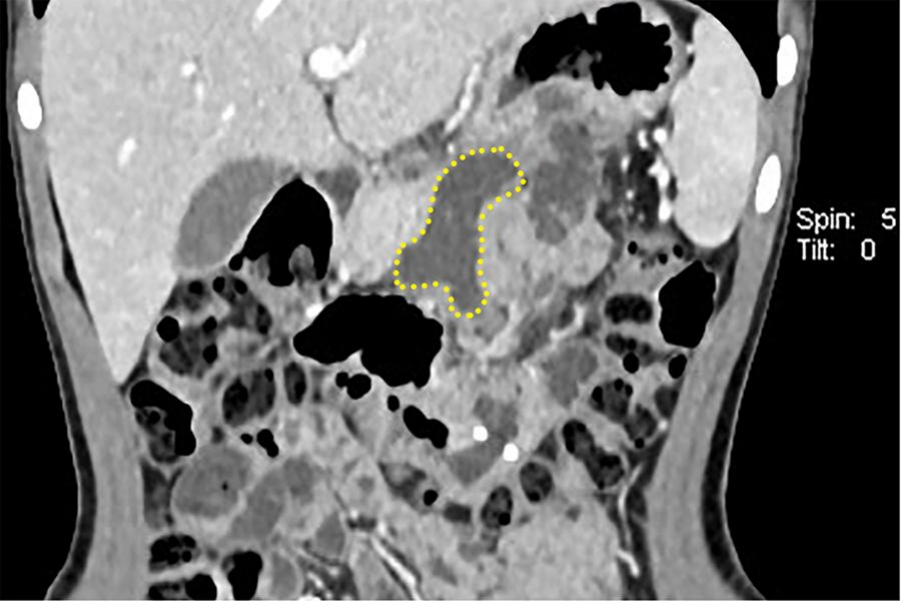
(Case 3): follow-up CT scan 11 days postoperatively shows localized fluid collection around the anastomosis (yellow circle).

**Figure 8:**
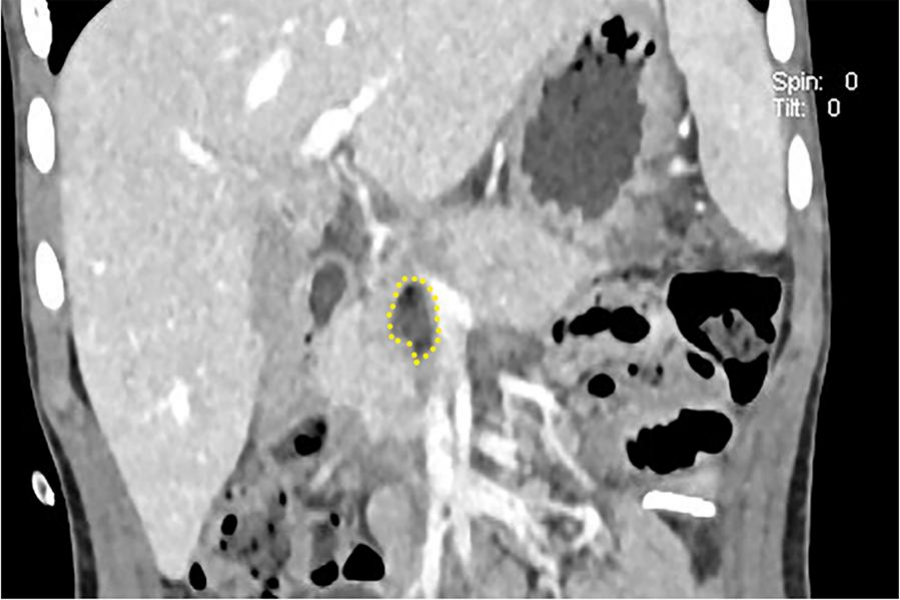
(Case 5): follow-up CT scan 9 days postoperatively shows a small amount of localized fluid collection around the anastomosis (yellow circle).

**Figure 9:**
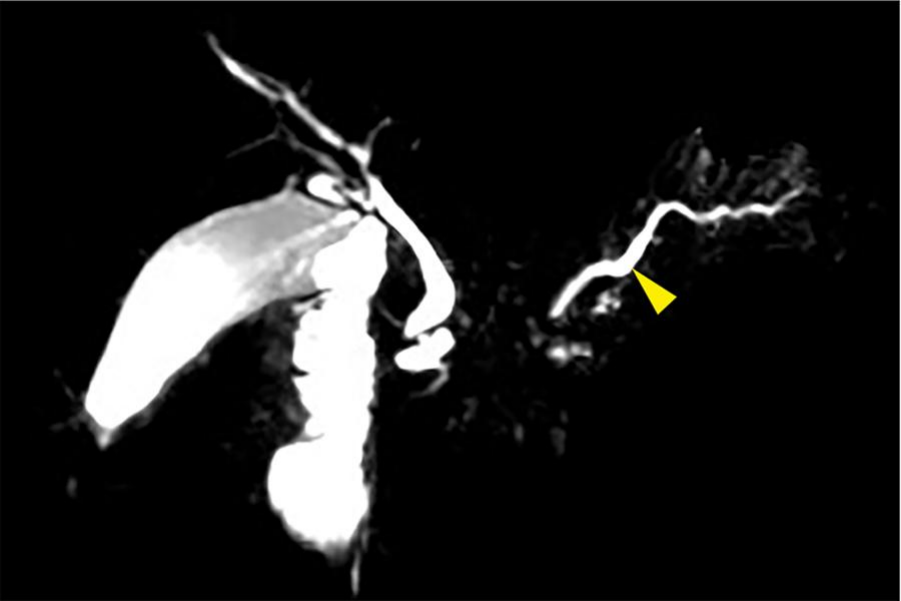
(Case 2): follow-up MRI/MRCP 25 months postoperatively shows mild local dilation of the pancreatic duct, with a maximum width of approximately 2.9 mm (yellow arrowhead).

**Figure 10:**
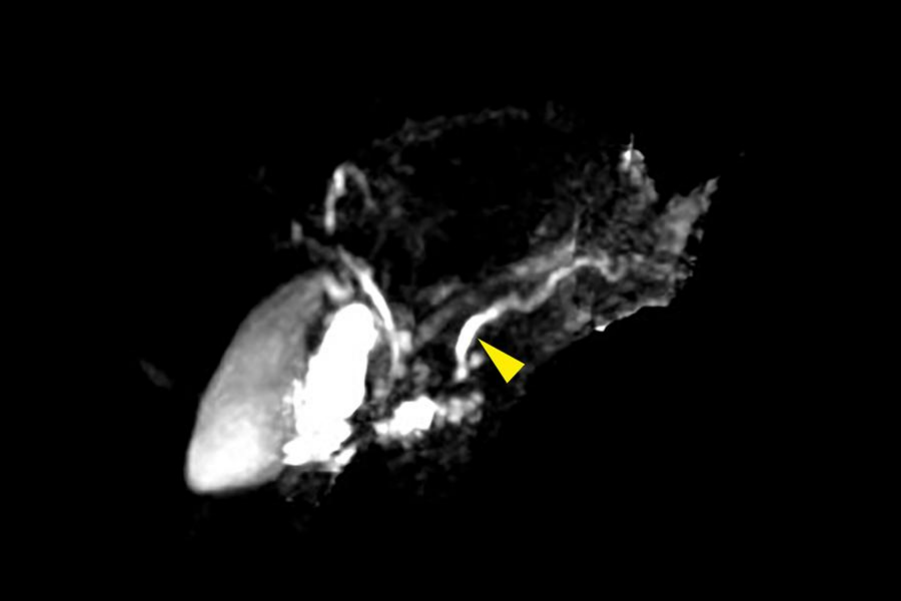
(Case 5): follow-up MRI/MRCP 4 months postoperatively shows mild dilation of the pancreatic duct, with a maximum width of approximately 2.3 mm (yellow arrowhead).

1. **Perioperative outcomes:** All 5 surgeries were completed successfully. Operative time ranged from 3 to 8 h (median, 5 h); the mean pancreaticojejunostomy time was 17.8 min. Intraoperative blood loss ranged from 20 to 100 mL (median, 50 mL); two cases required intraoperative transfusion of 1 unit of packed red blood cells.
2. **Early postoperative complications:** No grade B or C clinically relevant postoperative pancreatic fistulas occurred (0%, 95% CI: 0.0%–52.2%). Two patients (Cases 3 and 5) developed localized perianastomotic fluid collections approximately 10 days postoperatively (Figures 7, 8); in one of these cases, amylase levels in the drain effluent transiently exceeded three times the upper limit of normal serum amylase, meeting the diagnostic criteria for a biochemical leak. These CT scans were performed as per our institutional surveillance protocol to assess anastomotic integrity, not because of symptoms. Neither patient exhibited clinical symptoms such as fever or abdominal pain; no specific interventions were performed, and the fluid collections resolved completely after 2–6 weeks of follow-up. Two other patients (Cases 3 and 4) experienced transient elevations in serum amylase (less than twice the upper limit of normal) around 10 days postoperatively, without significant accompanying symptoms (abdominal pain, vomiting, or fever). According to the IAP criteria described in the Methods section, these patients did not meet the definition of postoperative acute pancreatitis because the amylase elevation was below the diagnostic threshold and imaging showed only localized perianastomotic changes without diffuse parenchymal involvementt (7). Therefore, these were managed as self-limiting localized inflammatory responses rather than clinical acute pancreatitis. Amylase levels spontaneously returned to normal within 2–3 weeks, and none progressed to severe pancreatitis. There were no cases of postoperative intra-abdominal hemorrhage, infectious complications, or mortality in this series. Abdominal drains were successfully removed in all patients between postoperative days 5 and 14 (median, 7 days). In Cases 3 and 5, drains were kept until POD 10–14 due to the fluid collection, which was managed expectantly without intervention.
3. **Long-term follow-up results:** At the last follow-up, all patients demonstrated normal growth and development curves. There were no new-onset cases of diabetes (fasting blood glucose and HbA1c remained normal), and no steatorrhea requiring pancreatic enzyme supplementation. Imaging follow-up revealed: In Case 2, the maximum pancreatic duct diameters at 3, 16, and 25 months postoperatively were 2.5, 3.4, and 2.9 mm, respectively (Figure 9); the patient remained asymptomatic with normal pancreatic parenchyma. In Case 5, the pancreatic duct measured 2.3 mm at 4 months postoperatively (Figure 10), with normal morphology and a patent anastomosis. The remaining three cases showed essentially normal pancreatic morphology without dilation of the main pancreatic duct. No tumor recurrence was observed (see Table 2 for details).

**Table 2:** Intraoperative, perioperative, postoperative conditions, and follow-up of 5 pediatric patients.

Case	Surgical procedure	Operative time (h)	Pancreaticojejunal anastomosis time (min)	Blood loss (mL)	Postoperative amylase		Perianastomotic fluid collection	Time to oral intake (POD)	Drain removal time (POD)	Follow-up time (M)	Long-term follow-up		
					Blood	Drainage fluid					Pancreatic duct diameter (mm)	Pancreatic morphology	Pancreatic endocrine and exocrine function
1	Whipple	5	20	20	N	N	No	5	7	92	N	N	Exocrine pancreatic insufficiency (steatorrhea)
2	DPPHR	5	16	100	N	N	No	5	7	25	2.9	N	N
3	LCP	8	20	100	↑	↑↑↑	Yes	7	14	16	1.4	N	N
4	PPPD	5.5	18	18	↑	N	No	6	7	49	1.5	N	N
5	Debridement + pancreaticocystojejunostomy	3	15	15	N	N	Yes	7	5	4	2.3	N	N

Whipple, pancreaticoduodenectomy; DPPHDR, duodenum-preserving pancreatic head resection; LCP, laparoscopic central pancreatectomy; PPPD, pylorus-preserving pancreaticoduodenectomy; POD, postoperative day; N, normal; ↑, mild persistent elevation of serum amylase after resuming oral intake [< 2× the upper limit of normal (ULN)]); ↑↑, postoperative (≥ 3 days) amylase concentration in any amount of drainage fluid > 3× ULN.

## Discussion

Pediatric pancreatic surgery, particularly digestive tract reconstruction involving pancreaticojejunostomy, presents significantly higher technical difficulty and risk compared to adult procedures. Previous studies report an overall postoperative pancreatic fistula (POPF) rate of approximately 3%–10.5% following pediatric pancreatic surgery (14, 15). Once severe complications such as POPF occur, they can not only trigger life-threatening massive hemorrhage, severe infection, and multiple organ dysfunction due to continuous stimulation by corrosive digestive enzymes, but also lead to progressive destruction of the residual pancreatic parenchyma, resulting in permanent exocrine and endocrine pancreatic insufficiency (16).

High-quality anastomotic technique is key to reducing POPF (17). Although considerable evidence-based data have been accumulated regarding anastomotic methods in adult pancreatic surgery, research focused on the pediatric population remains relatively limited. In current single-center reports with small sample sizes, anastomotic approaches are largely extrapolated from adult experience, with duct-to-mucosa pancreaticojejunostomy, end-to-end pancreatic stump-to-jejunum anastomosis, and invaginated pancreaticojejunostomy all having been reported (16, 18, 19). However, due to the soft and fragile texture of the pediatric pancreas and the small diameter of the main pancreatic duct (MPD), the applicability of duct-to-mucosa pancreaticojejunostomy is limited. The other two techniques have also been associated in existing reports with complex procedures, prolonged operative time, and higher risks of POPF (20).

Recent studies indicate that the modified stent-free Chen's U-suture technique can not only simplify surgical manipulation and shorten operative time but also reduce the incidence of POPF, proving especially suitable for pancreases with soft texture and narrow MPDs (21). Our observed 0% CR-POPF rate (95% CI: 0.0%–0%.2%) in this high CR-POPF rate (95% CI: 0surgical manipulation and shorten operative time but also reduce the incidence of POPF, proving esp (1), and appears favorable against the 14–31.5% rates reported for other pediatric PJ techniques (16, 20, 22). Accordingly, we applied this technique for pancreaticojejunostomy in five pediatric patients meeting surgical indications at our institution. Clinical observations demonstrate that the technique exhibits promising safety and feasibility in the pediatric population.

In this case series, the MPD was either not visualized on preoperative imaging or measured ≤2 mm in diameter. Combined with thermal injury generated during pancreatic parenchymal transection using an ultrasonic scalpel, as well as factors such as pancreatic trauma and inflammatory response, intraoperative identification of the MPD proved difficult. This challenge was particularly pronounced in patients with severe duodenal ampullary injury and pancreatic trauma (Cases 4 and 5), where leakage of pancreatic juice, bile, and gastrointestinal fluids caused severe corrosion and inflammation around the pancreatic gland and peripancreatic tissues, rendering the tissue fragile and prone to bleeding, further complicating identification of the MPD at the transection surface. In such cases, traditional end-to-end or invaginated anastomoses carry a high risk of pancreatic tissue tearing or stump ischemia.

We employed the modified stent-free Chen's U-suture technique, which is analogous to driving two rows of “stakes” into the pancreas. This approach reduces the risk of tissue tearing, achieves a double-layered invagination effect upon tightening the sutures, requires minimal mobilization of the pancreatic stump (thereby lowering the risk of compromising blood supply to the stump), and simplifies the surgical procedure, necessitating only 6–8 continuous sutures. In existing adult literature, this method has demonstrated significantly shorter anastomosis time compared to duct-to-mucosa pancreaticojejunostomy (23). This advantage stems from its simplified operational workflow based on continuous transpancreatic suturing, making it particularly suitable for cases with soft pancreatic texture. In our case series, the mean time required solely for pancreaticojejunostomy was only 17.8 min, substantially saving operative time, with results meeting expectations.

Following the adoption of this anastomotic technique, no clinically relevant pancreatic fistulas (Grade B/C) occurred in this cohort. Two pediatric patients with preserved pancreatic head remnants (one following mid-pancreatectomy and the other post-trauma) developed localized perianastomotic fluid collections approximately 10 days postoperatively. In one case, the drain fluid amylase level met the criteria for a biochemical leak; in the other, testing was not possible as the drain had already been removed. In contrast, two pediatric patients who underwent pancreaticoduodenectomy (with complete resection of pancreatic tissue on the pancreatic head side) and one patient with minimal preserved pancreatic head tissue did not exhibit similar signs of fluid collection postoperatively.

Furthermore, two pediatric patients in this series (Cases 3 and 4) exhibited a transient mild elevation in serum amylase around 10 days postoperatively, which spontaneously returned to normal after 2–3 weeks. The cause of these self-limited amylase elevations is unclear. As defined by the 2025 IAP criteria (see Methods), these events do not constitute postoperative acute pancreatitis (7). Similar biochemical phenomena have been described after pediatric pancreatic surgery without clinical consequences (22).

In this cohort, no severe complications related to main pancreatic duct ligation occurred in the absence of pancreatic duct stenting. The invaginated structure of the Chen's U-suture technique may provide a multi-channel compensatory drainage mechanism, and all patients received fasting and somatostatin analogs postoperatively. However, these observations are hypothesis-generating only.

Following pediatric pancreatic surgery, the core long-term concern is the preservation of endocrine and exocrine functions. With a median follow-up of 25 months in this cohort, no patient developed new-onset diabetes or exocrine insufficiency requiring clinical intervention. This indicates that the modified stent-free Chen's U-suture technique can effectively maintain long-term function while preserving sufficient pancreatic volume and blood supply. This benefit stems from its relatively conservative approach to handling the pancreatic stump and minimal disruption to pancreatic parenchymal blood supply.

Notably, Case 2 developed distal pancreatic duct dilation postoperatively (maximum diameter 2.9 mm). Mild asymptomatic pancreatic duct dilation was observed in two patients, but neither showed progressive worsening, parenchymal atrophy, or clinical symptoms. Longer-term follow-up is warranted to monitor for any progression. Additionally, in Case 5 (post-trauma), the pancreatic duct diameter was 2.3 mm at 4 months postoperatively, which is at the upper limit of the normal range, also necessitating long-term observation.

## Limitations

Although this study preliminarily demonstrates the potential of the modified stent-free Chen's U-suture technique in pediatric pancreatic surgery, several limitations exist. First, as a single-center retrospective study, the sample size is small (only 5 cases), resulting in a wide 95% confidence interval for the CR-POPF rate (0.0%–52.2%), and the follow-up duration varies significantly (4–92 months), which may affect the accuracy of assessing long-term complications such as progression of pancreatic duct dilation or decline in pancreatic function. Second, the lack of a control group (e.g., traditional duct-to-mucosa anastomosis or pancreatic duct stenting groups) for direct comparison limits the generalizability of the conclusions. Third, drain fluid was tested only for amylase; triglycerides and bilirubin were not measured routinely, which may have underdetected chyle or biliary leaks. Fourth, there is currently a lack of standardized diagnostic criteria for postoperative acute pancreatitis in the pediatric population, which may lead to ambiguity in reporting and interpreting mild postoperative amylase elevations and imaging changes. The 2025 IAP guidelines have highlighted that although the diagnostic criteria for acute pancreatitis in children are the same as in adults, their accuracy is lower and no definition has been validated for the postoperative setting (7). Future research should establish pediatric-specific criteria for post-pancreatectomy acute pancreatitis.Therefore, future multi-center, prospective studies with extended follow-up periods and comprehensive drain fluid analysis are needed to more comprehensively validate the long-term value of the modified stent-free Chen's U-suture technique in pediatric pancreatic surgery.

## Conclusion

This study provides preliminary evidence that the modified stent-free Chen's U-suture technique is feasible and shows promising safety signals and efficacy in the digestive tract reconstruction following pediatric pancreatic head-neck tumors, grade III pancreatic trauma, and complex iatrogenic injuries. In this small cohort, no CR-POPF occurred (0%, 95% CI: 0.0%–52.2%). Even without routine pancreatic duct stenting, the incidence of postoperative pancreatic fistula appears manageable in this limited experience, and the long-term pancreatic endocrine and exocrine functions are generally preserved. This surgical method is operationally simplified and has a short anastomosis time. It may be particularly suitable for pediatric cases with soft pancreatic tissue and thin pancreatic ducts. For surgeries that retain the pancreatic head side stump, attention should be paid to the impact of stump handling techniques on postoperative effusion; for the self-limiting elevation of serum amylase and mild dilation of the distal pancreatic duct that occur after surgery, it is recommended to strengthen long-term imaging follow-up. Future studies with a larger sample size are needed to further verify the advantages of this surgical method. Larger studies are needed to confirm these preliminary findings.

## Data Availability

The original contributions presented in the study are included in the article/Supplementary Material, further inquiries can be directed to the corresponding author.
